# Twenty-Eighth Annual Report on the State of the Asylum for the Relief of Persons Deprived of Their Reason

**Published:** 1845-05

**Authors:** 


*Twenty-Eighth Annual Report on the state of the Asylum for the Relief of Persons deprived of their Reason.* Published by direction of the Contributors. Philadelphia, Third month, 1845.

This excellent Institution is located in a fine, healthy district of country, about six miles north of Philadelphia, and in the vicinity of the borough of Frankford. It is owned and governed by an Association, the members of which are confined to those of the Society of Friends. It was originally designed exclusively for the relief of members of that religious denomination, but the liberality of the proprietors has extended the privilege to respectable individuals out of the pale of the Society, when there is room in the buildings for their accommodation, without interfering with the admission of those contemplated by its establishment.

The immediate government of the Institution is confided to a Board of Managers selected from among the Contributors. Dr. Charles Evans is the Attending Physician, and Dr. Joshua H. Worthington, Resident Physician.

| | | |
|---|---|---|
| The number of those in the Asylum at the time of the last report, was - - - | 52 | 100 |
| Received since, - - - - - - | 48 | |
| Discharged or died, - - - - - - | 42 | 100 |
| Remaining, - - - - - - | 58 | |

Of the forty-two patients discharged, there were

| | |
|---|---|
| Restored, - - - | 25 |
| Convalescent, - - | 2 |
| Much improved, - - | 2 |
| Improved, - - - | 7 |
| Stationary, - - - | 5 |
| Died, - - - | 1—42 |

Of the fifty-eight remaining, there are

| | |
|---|---|
| Restored, - - - | 3 |
| Much improved, - - | 5 |
| Improved, - - - | 8 |
| Unimproved, - - - | 42—58 |

Of the patients discharged "restored," eleven were in the Asylum not exceeding three months; seven from three to six months; and seven from six months to a year.

The average duration of residence in the Institution, for the twenty-five cases restored, was four months and nineteen days.

